# Effects of Weekly Supplementation of Cholecalciferol and Calcifediol Among the Oldest-Old People: Findings From a Randomized Pragmatic Clinical Trial

**DOI:** 10.3390/nu11112778

**Published:** 2019-11-15

**Authors:** Carmelinda Ruggiero, Marta Baroni, Vittorio Bini, Annalisa Brozzetti, Luca Parretti, Elisa Zengarini, Maria Lapenna, Pierluigi Antinolfi, Alberto Falorni, Patrizia Mecocci, Virginia Boccardi

**Affiliations:** 1 Gerontology and Geriatrics Institute, Department of Medicine, University of Perugia, Santa Maria della Misericordia Hospital, 1 06123 P.le Menghini, Italy; martabaroni@libero.it (M.B.); luca.parretti@libero.it (L.P.); marialapenna89@gmail.com (M.L.); patrizia.mecocci@unipg.it (P.M.) virginia.boccardi@unipg.it (V.B.); 2 Department of Medicine, Section of Internal Medicine and Endocrine and Metabolic Sciences, University of Perugia, Santa Maria della Misericordia Hospital, 1 06123 P.le Menghini, Italy; vittorio.bini@unipg.it (V.B.); a.brozzetti@libero.it (A.B.); alberto.falorni@unipg.it (A.F.); 3 Geriatric Medicine and Geriatric Emergency Care, Italian National Research Center on Aging (IRCCS INRCA), 60127 Ancona, Italy; el.zengarini@hotmail.it; 4 Division of Orthopedics and Trauma Surgery, Santa Maria della Misericordia Hospital, 06123 Perugia, Italy; pantinolfi@gmail.com

**Keywords:** calcifediol, cholecalciferol, bone markers, oldest-old, polypharmacy, sarcopenia

## Abstract

Vitamin D inadequacy is pervasive in the oldest-old. Many vitamin D metabolites are available for supplementation, their effects on the recovery of adequate serum levels remain unknown. We investigate the effects of supplementation with cholecalciferol (D3) and calcifediol (25D3) on serum levels of 25(OH)D, 1-25(OH)D, bone and inflammatory markers, ultimately identifying clinical predictors of successful treatment. Sixty-seven oldest-old individuals were randomized to weekly administration of 150 mcg of 25D3 or D3, from hospital admission to 7 months after discharge. Supplementation of 25D3 and D3 were associated with increasing serum levels of 25(OH)D (*p* < 0.001) and 1-25(OH)D (*p* = 0.01). Participants on 25D3 experienced a steeper rise than those on D3 (group*time interaction *p* = 0.01), after adjustment for intact parathyroid hormone (iPTH) levels the differences disappeared (intervention*iPTH interaction *p* = 0.04). Vitamin D supplementation was associated with a decreasing trend of iPTH and C-reactive protein (CRP) (*p* < 0.001). Polypharmacy and low handgrip strength were predictors of failure of intervention, independent of vitamin D metabolites. In conclusion, D3 and 25D3 supplementation significantly increase vitamin D serum levels in the oldest-old individuals, with a tendency of 25D3 to show a faster recovery of acceptable iPTH levels than D3. Polypharmacy and low muscle strength weaken the recovery of adequate vitamin D serum levels.

## 1. Introduction

Low serum levels of vitamin D are pervasive in older people, especially among those with fragility fractures [1]. Falls and fractures are causally related to low serum 25-hydroxyvitamin D [25(OH)D], leading to decreased intestinal calcium absorption, increased intact parathyroid hormone (iPTH) secretion and bone resorption, impaired muscle function and bone strength. Worldwide, fragility fractures represent a challenge for the healthcare services, with rates of ~30% for hip fractures and ~40% for vertebral fracture in the oldest-old people, especially women [2].

Serum levels of 25(OH)D ≤ 10–12 ng/mL identifies a vitamin D deficiency, which is extremely risky for bone and muscle health in the general population, even moreso in seniors, who are at risk of falls and fractures [3]. Serum levels of 25(OH)D ≥ 20 ng/mL are likely adequate in the general population [4], but a threshold of 25(OH)D > 30 ng/mL is advisable for individuals at high risk for fracture, especially if anti-fracture treatment is used [5]. Serum levels of 25(OH)D > 30–33 ng/mL improve the response of postmenopausal osteoporotic women to bisphosphonates. Furthermore, studies point to 25(OH)D between 22–30 ng/mL as the optimal therapeutic range with respect to fall prevention in seniors who had a prior fall, while 25(OH)D concentrations below 20–22 ng/mL (53 nmol/L) and above 45–50 ng/mL (112–125 nmol/L) are associated with increased risk of falling [6].

Older people with low serum vitamin D need supplements to meet their requirements [7]. Sunlight or artificial UVB light exposure and dietary intake of vitamin D are not real options to correct the deficiency in the older sub-groups of the population [8]. The therapeutic strategies to deal with vitamin D deficiency include cholecalciferol (D3) the most used supplement in Europe, and ergocalciferol (D2) most frequently used in North America. Comparative studies have shown that daily supplementation with D2 is only about 70% as potent as D3 in raising serum 25(OH)D in deficient adults [9]. In healthy adults and seniors, daily low-dose D3 supplementation (800 UI) requires almost 2 years to achieve the optimal 25(OH)D threshold, while it may be reached within 42 days by using daily high-dose of D3 supplementation (from 1800 to 4000 UI) [10]. Long lasting research is trying to identify the ideal high-dose D3 supplementation and many concerns have risen with D3 bolus, given the mounting rate of falls, fractures and alterations of bone turnover markers [11]. Calcifediol (25D3) is a biochemical product different from D3, but is likely an alternative option to D3. Comparative studies show that 25D3 has hydrophilic properties and rapidly increases blood serum 25(OH)D levels; 25D3 stays longer in the blood because of longer half-life (15 to 18 days) after oral administration. Dosage and timing of 25D3 are markedly different from those for D3 supplementation, because 25D3 can directly bind and activate the vitamin D receptor (VDR), and has a more potent action to suppress iPTH levels.

To the best of our knowledge, scant evidence exists on the efficacy of a comparable dose of 25(OH)D and D3 to meet the serum requirements of 25D3 in oldest-old seniors. The aim of this study was to investigate the effects of 25D3 and D3 supplementation, by using a dose of 150 mcg/weekly, on serum levels of 25(OH)D, 1-25(OH)D, iPTH and bone turnover markers in oldest-old persons. The effects of 25D3 and D3 supplementation on C Reactive Protein (CRP) was explored and, ultimately, the predictors of the therapeutic success of interventions, defined as the best target levels for 25(OH)D, iPTH, CRP and albumin at the end of follow-up, were investigated.

## 2. Materials and Methods

### 2.1. Study Design and Sample

A randomized controlled pragmatic trial was conducted among community-dwelling women and men, aged >75 years, consecutively admitted to geriatric acute care ward of the S. Maria Misericordia Hospital, Perugia, Umbria (Italy). This is a teaching hospital meeting the acute care needs of almost 900,000 inhabitants of the Umbria region of Italy, where one in four individuals (24%) is over 65 years. Participants were excluded if they were on treatment with vitamin D, multivitamins, calcium supplements, anti-fracture drugs or steroids, or they suffered from fragility fractures within 6 months, cancer within 5 years, hyperparathyroidism, hypercalcemia, hypercalciuria, hypophosphatemia, Paget’s disease, chronic renal failure, nephrolithiasis, bowel inflammatory diseases, bowel resection, malabsorption syndrome, including celiac and Crohn’s diseases, liver disease, excessive alcohol use, tuberculosis or sarcoidosis. A trained physician examined all participants. Diseases were ascertained according to pre-established criteria that combined information from physician diagnosis, medical records, clinical examination, and blood clinical tests. Exclusion criteria applied also if they underwent major surgical within 6 months, were unable to walk outdoors before admission, were bedridden, nursing home residents at the time of admission or become eligible for nursing home placement during the course of the hospital stay, or if they participated in other clinical studies. Sixty-seven individuals agreed to participate in the study during an enrollment period of 12 months. In this period, 932 oldest-old patients were consecutively admitted to the ward, 233 were eligible to the study and 104 refused to give informed consent and/or to participate in the study due to several difficulties, mainly a lack of transportation for attending the follow-up and refusal of additional blood withdrawals. In addition, 65 participants exited the study after randomization because some exclusion criteria were discovered during the hospital stay. Individuals who passed eligibility criteria and signed written informed consent were randomly assigned to intervention groups. Participants randomized to group A received a weekly dosage of 150 mcg 25D3, while those in group B received a weekly dosage of 150 mcg D3 the day after admission. At the time of hospital admission, the on call resident, in collaboration with the geriatrician on call, had the responsibility for the randomization by using the coin-flipping procedure. The randomization was performed after verifying the exclusion criteria based on the previous clinical documentation. At the time of randomization, the resident and the geriatrician on call were blind to the baseline serum levels of 25(OH)D and biochemical parameters of bone metabolism and they were in charge to prescribe the intervention on the clinical chart. Noteworthy, participants underwent assessment for comorbidities by the resident and geriatrician in charge during hospital stay; thereby, participants who revealed to meet exclusion criteria even after randomization and taking vitamin D automatically lapsed the study. Participants started to take D3 or 25D3 supplements orally only after the baseline blood withdrawals for the study were performed, and until 8-months from baseline. Prescriptions of supplements were performed in accordance with study protocol and drug indications; they were recorded in the clinical chart during the hospital stay and participants received D3 or 25D3 supplement by the nursing staff during the daily rounds. The dosage of 25D3 was chosen to compare the current standard for 25D3 (21 mcg/day), which offer an optimal balance of efficacy and safety [12,13] according to a review of the literature. The dose for D3 supplementation was the standard (20 mcg = 800 UI) at the time of protocol approval in Italy. During the hospital stay, participants underwent blood withdrawals at 5 and 10 days from intervention. Participants received a prescription of 150 mcg D3 or 25D3 weekly, according with allocation group, written in the hospital discharge letter. The ambulatory follow-up visits were scheduled at 2 and 7 months from discharge and underlined the discharge letter. The study was conducted in accordance with the declaration of Helsinki and approval was obtained from the General Director of the Santa Maria Misericordia Hospital, Perugia, Umbria (Italy) with registration number 757/5.6.2014, and the Ethics Committee of the Healthcare Agencies of Umbria Region of Italy (CEAS Umbria), with registration number 2121/13. Written informed consent was obtained from all patients participating in the study.

### 2.2. Data Collection and Biochemical Parameters

Fasting blood and urine samples were obtained between 6:00 AM and 7:00 AM at all time points during the hospital stay, between 7:30 and 8:30 during outpatient follow-up times. Fasting blood samples were centrifuged and the sera were stored at −80 °C until the measurements were performed. All subjects underwent a careful clinical examination and a detailed interview by a geriatrician, a proxy received an additional interview to confirm collected data. Demographic, anthropometric, behavioral-related data and clinical parameters were gathered from the clinical records and by using standardized questionnaires. Body mass index (BMI) was calculated by dividing the weight in kilograms by the square of height in meters. Physical function was assessed using standardized questionnaire and performance measures. Each subject was interviewed regarding their disability in basic activities of daily living (ADL) [14] and in instrumental activities of daily living (IADL) [15]. The number of reported disabilities was used for the present study. Cognitive functions were evaluated by using MiniMental State Examination (MMSE), that is the most commonly used scale. Frailty syndrome was defined according to the Canadian Study of Health and Aging Clinical Frailty Scale (CSHA-CFS) with a threshold > 4 to identify frail older people [16,17]. Muscle strength was measured by using grip strength (GS) test performed using a North Coast Medical hand dynamometer. Patients were seated with the wrist in a neutral position and the elbow flexed 90°. In case a subject was unable to sit, GS was assessed lying at 30° in bed with the elbows supported. The highest value of two consecutive measurements obtained with the dominant hand was used in the analyses. Diseases were ascertained according to pre-established criteria that combined information from physician diagnosis, medical records, clinical examination and blood clinical tests. Kidney function was estimated by creatinine-based glomerular filtration rate, using the simplified Modification of Diet in Renal Disease equation [18]. Serum levels of 25(OH)D and 1-25(OH)D were measured by radioimmunoassay (RIA kit; DiaSorin, Stillwater, MN, USA). Serum iPTH levels were measured by an immunoenzymatic method (Access; Beckman Coulter INc., Fullerton, CA, USA). Serum total phosphorus, calcium and albumin were measured by an automated chemistry analyzer. Albumin-corrected calcium was calculated as calcium + [0.8 × (4-serum albumin)]. The bone-specific isoenzyme of alkaline phosphatase (BAP) was measured by IDS-iSYS Ostase^®^ BAP Assay (Beckman Coulter, Inc). The C-terminal telopeptides of type I collagen was measured by IDS-iSYS Automated System CTx-I (CrossLaps^®^) Assay (Immunodiagnostic Systems Limited, UK). Serum C-reactive protein (CRP) was measured using immunoturbidimetric method for quantitative determination (Beckman Coulter AU Analyzers). The index of the therapeutic success of intervention was a score based on the sum of points attributed to the individual serum levels of 25(OH)D, iPTH, CRP and albumin, attained at the end of the study period. According with safety thresholds, a score of 2 was given if at the end of the study the individual levels of 25(OH)D > 30 ng/mL, iPTH < 60 pg/mL, CRP < 1.5 mg/dl, and albumin > 4.5 g/dl. A score of 1 was given if levels of 25(OH)D were between 20 and 30 ng/mL, iPTH between 60 and 85 pg/mL, CRP between 3.5 and 1.5 mg/dl, and albumin between 3.5 and 4.5 g/dl. A score of 1 was attributed if levels of 25D3 < 20 ng/mL, iPTH > 85 pg/mL, CRP > 3.5 mg/dl, and albumin < 3.5 g/dl. The total score of the index ranged from 0 to 8, and a cut-off > 6 defined the therapeutic success of the intervention.

### 2.3. Statistical Methods

#### 2.3.1. Sample Size and Missing Data

A power analysis was conducted to determine the number of participants needed in this study using G-Power version 3.1.9.2, 2014 [19]. A sample size of 32 for each group at study achieved 80% power to detect differences among the means versus the alternative of equal means using an F test at a 0.05 significance level. The size of the variation in the means is represented by 0.20 of their standard deviation [20]. Multiple imputations (MI) was used as the method to account for missing data at both baseline and follow-up. A chained equation model was applied [21]. All missing data were imputed separately by trial group. The percentage of missing data served as the base of the number of imputations, as a rule of thumb [22]. We used a conservative 40 imputations in all MI analyses, which were conducted using XLSTAT 2016 (Data Analysis and Statistical Solution for Microsoft Excel. Addinsoft, Paris, France, 2016).

#### 2.3.2. Statistical Analysis

The Shapiro-Wilk test was used to assess the normal distribution of variables and the Mann–Whitney’s U-test was used for comparisons of non-normally distributed continuous variables at the start of the clinical trial. Frequencies of qualitative data (sex) were analyzed using the χ2 test with Yates’ continuity correction. Comparisons within and between groups over time were analyzed by mixed model repeated measures analysis of variance (RM ANOVA), with Bonferroni’s correction for post-hoc comparisons. “Time” (variable values at 0, 5, 10, 60 and 210 days) and “group” (therapy arms) were considered as within-subjects and between-subjects factors, with five “time” and two “group” levels, respectively. The relationship between the above variables and time was tested by linear regression analysis, and their slopes were compared by the Student t-test. Because of non-normally distribution of residuals, data were transformed by using Box–Cox method [23] to better approximate Gaussian distribution of residuals which was verified by the Shapiro-Wilk test. Bivariate and multivariate logistic regression models were fitted for the prediction of therapeutic success, incorporating all the variables that showed a *p*-value ≤ 0.25 in bivariate analysis as explanatory variables [24]. To decrease the over-fit bias and internally validate our results, all bivariate and multivariate regressions underwent 200 bootstrap resamples and the goodness-of-fit of logistic models were checked using Hosmer and Lemeshow test. Odds ratios (ORs) with 95% confidence intervals were also calculated. Predictor variables were tested for collinearity and dropped from the regression models, as appropriate. All statistical analysis was performed by using IBM-SPSS^®^ version 25.0 (IBM Corp., Armonk, NY, USA, 2017). In all analyses, a two-sided *p*-value <0.05 was considered statistically significant.

## 3. Results

### 3.1. Baseline Characteristics of Participants

Table 1 presented the baseline characteristics of the participants according to group intervention. Overall, participants were more likely to be women, equally distributed in both groups (women 64% versus 60%; *p* = 0.9), with a median age over 80 years (83.5 versus 82.0; *p* = 0.2). At hospital admission, participants showed cognitive functions at MMSE substantially preserved, they reported independence in ADL and ability to perform several IADL, they appeared clinically vulnerable at the CSHA scale (4 versus 4; *p* = 0.9). Participants were similar between group A and B as regard of BMI (26.8 versus 27.5; *p* = 0.1), MNA (24.5 versus 24.0; *p* = 0.2), sex-specific handgrip test (women: 22.8 versus 20.3, *p* = 0.8; men: 28.8 versus 28.0; *p* = 0.3), systolic, diastolic blood pressure and heart rate, comorbidity (7 versus 7; *p* = 0.6) and polypharmacy (6 versus 5; *p* = 0.2). Serum levels of 25(OH)D were overlapped between group A and B (10 versus 10; *p* = 0.7), as the serum levels of 1-25(OH)D (27 versus 22; *p* = 0.4). Vitamin D deficiency was found in more than 60% of participants (59% versus 66%; *p* = 0.5), and vitamin D insufficiency in more than 90% (88% versus 93%; *p* = 0.4), in group A and B, respectively. Several biochemical parameters, including CRP (2.9 versus 3.1, *p* = 0.6), creatinine (0.9 versus 0.8, *p* = 0.1), calcium (8.8 versus 8.8, *p* = 0.2), phosphorus (3.5 versus 3.4, p = 0.3), magnesium (2 versus 2.1, *p* = 0.3), CTx-I (0.8 versus 0.7, *p* = 0.2) and ALPs (9.1 versus 9.2, *p* = 0.9), were almost superimposed between groups. iPTH levels were higher in participants belonging to group A then those in group B (104 pg/mL versus 50 pg/mL; *p* = 0.002). Overall, 37% of participants suffered from hyper-parathyroidism secondary to vitamin D inadequacy with higher prevalence in A as compared to group B (*p* = 0.007). The difference between groups was completely due to chances, given that randomization preceded the blood withdrawal for serum levels of bone markers.

### 3.2. Sample Size at Baseline and During Follow-Up Times

All participants were alive at the end of the study period, with 72% of those belonging to group A and 67% of those belonging to group B contributing over the entire study period. Overall, participants contributed with 87 (88%) and 85 (83%) serum evaluations over the in-hospital period, and with 43 (77%) and 42 (84%) serum evaluations over the outpatient period, respectively, in group A and B.

### 3.3. Longitudinal Effects of 25D3 or D3 Intervention on Serum Biochemical Parameters

**Figure 1:**
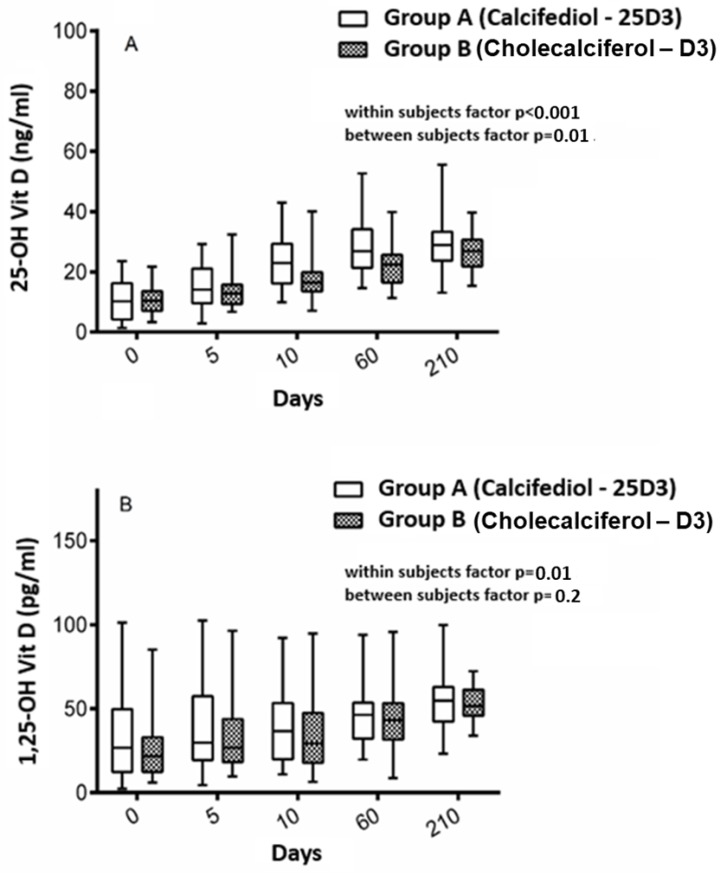
Serum levels of the metabolites of vitamin D over time among participants grouped according to the type of intervention. Panel **A** shows the serum levels of 25(OH)D over time among participants belonging to group A, those receiving calcifediol, and group B, those receiving cholecalciferol. Panel **B** shows the serum levels of 1-25(OH)D over time among participants belonging to group A, those receiving calcifediol, and group B, those receiving cholecalciferol.

**Figure 2:**
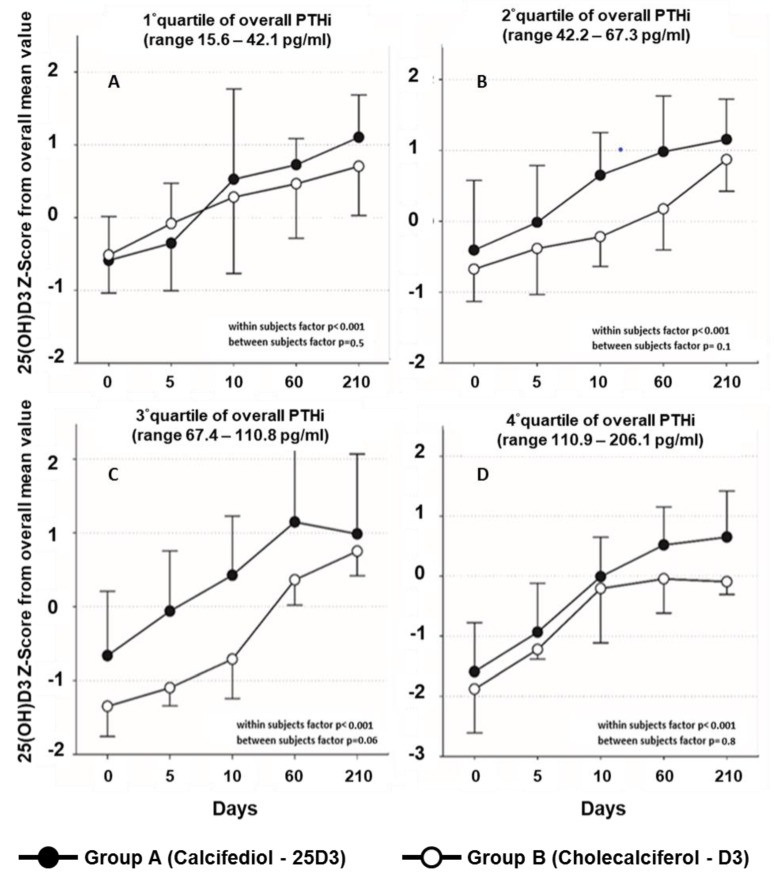
Mean values of serum levels of 25(OH)D, (expressed as a number of SD from the overall mean value), over time among participants grouped according to the type of intervention and stratified by quartiles of overall basal iPTH levels. Panel **A** shows the mean values of serum 25(OH)D levels over time among participants within the lowest quartile of iPTH levels and belonging to group A, those receiving calcifediol, and to group B, those receiving cholecalciferol. Panel **B** shows the mean values of serum 25(OH)D levels over time among participants in the second quartile of iPTH levels and belonging to group A, those receiving calcifediol, and to group B, those receiving cholecalciferol. Panel **C** shows the mean values of serum 25(OH)D levels over time among participants within the third quartile of iPTH levels and belonging to group A, those receiving calcifediol, and to group B, those receiving cholecalciferol. Panel **D** shows the mean values of serum 25(OH)D levels over time among participants within the highest quartile of iPTH levels and belonging to group A, those receiving calcifediol, and to group B, those receiving cholecalciferol.

In both groups, serum 25(OH)D levels increased persistently over time (*p* < 0.001), with a steeper slope in participants belonging to group A as compared with those belonging to group B (group*time interaction *p* = 0.01) (Figure 1, panel A). Supplementation with 25D3 acted faster to rise serum 25(OH)D levels compared to D3, especially within the 10 days of hospital stay (12 ng/mL versus 7 ng/mL; *p* < 0.001). At the end of the study, serum 25(OH)D increased on average by 19 ng/mL in participants belonging to group A and by 16 ng/mL in those belonging to group B (*p* = 0.5). Participants belonging to group A tended to reach the target threshold of 25D3 > 30 ng/mL more then those on group B (44.1% versus 33.3%, *p* = 0.5) at the end of follow-up. An interaction factor between vitamin D supplementation and baseline levels of iPTH was found (*p* = 0.04); then, slopes of increasing 25(OH)D levels became almost superimposed between groups by quartiles of basal iPTH levels (Figure 2). Participants experienced a statistically significant increase of serum levels of 1-25(OH)D (*p* = 0.01) over time, without differences between groups (*p* = 0.2) and without evidence for an interaction factor time*intervention (Figure 1, panel B).

**Figure 3:**
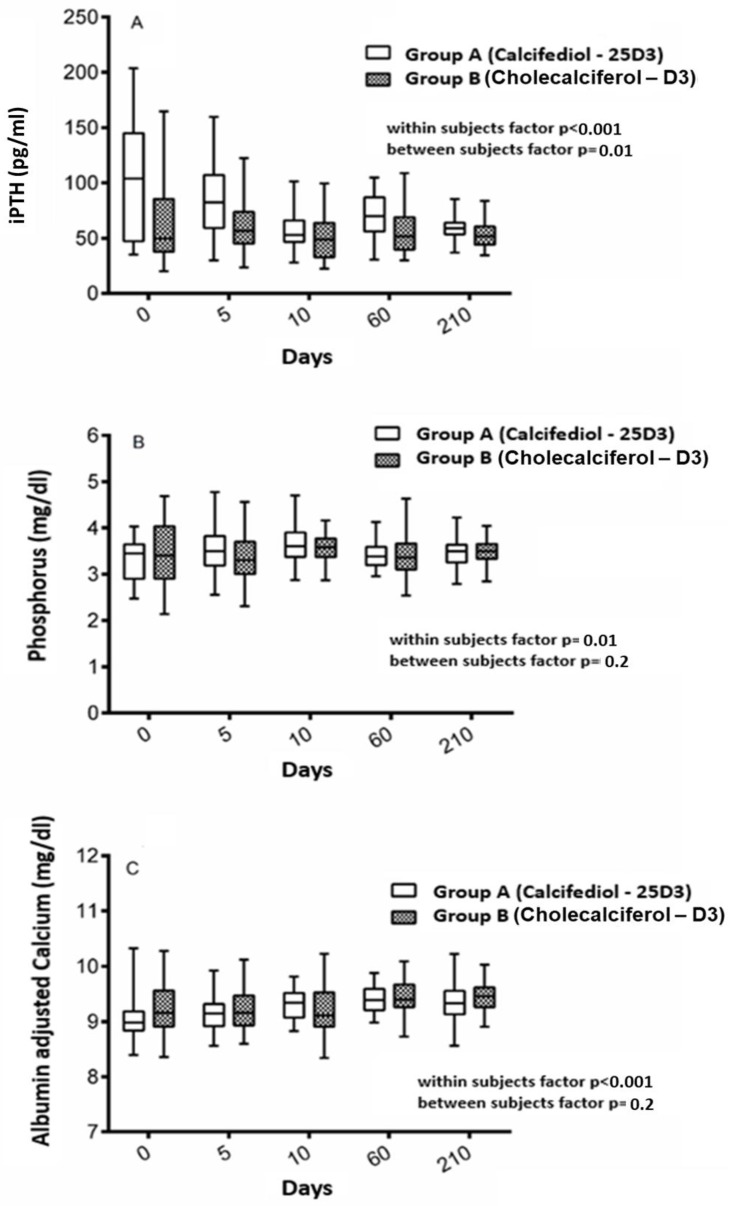
Serum levels of markers of bone metabolism over time among participants grouped according to the type of intervention. Panel **A** shows the serum levels of iPTH over time among participants belonging to group A, those receiving calcifediol, and group B, those receiving cholecalciferol. Panel **B** shows the serum levels of phosphorus over time among participants belonging to group A, those receiving calcifediol, and group B, those receiving cholecalciferol. Panel **C** shows the serum levels of albumin adjusted calcium over time among participants belonging to group A, those receiving calcifediol, and group B, those receiving cholecalciferol.

Consistent with changes of serum 25(OH)D levels, iPTH levels decreased over the study period (*p* < 0.001) in both groups, by showing a steeper decline among participants belonging to group A as compared to that found in those belonging to group B (group*time interaction *p* < 0.001) (Figure 3, panel A). For the entire study period, the iPTH levels declined from 99.6 ± 51.2 to 58.9 ± 13.9 pg/mL (average decline of 5.8 pg/mL per month) in group A, and from 64.1 ± 38.2 to 53.7 ± 13.6 pg/mL (average decline of 1.5 pg/mL per month) in group B, with a statistically significant difference between groups (*p* = 0.01). Although a difference between groups was formerly present at the beginning of the study (Table 1), the rate of participants reaching iPTH < 60 pg/mL tended to be similar in group A as compared to group B (61.8% versus 75.8%, p 0.3). Along these lines, serum calcium slightly increased over time within individuals (*p* < 0.001) with a similar extent between groups (*p* = 0.2) (Figure 3, Panel C). Likewise, serum phosphorus showed slightly positive changes over time within individuals (*p* = 0.01), and without difference between groups (*p* = 0.2) (Figure 3, Panel B).

**Figure 4:**
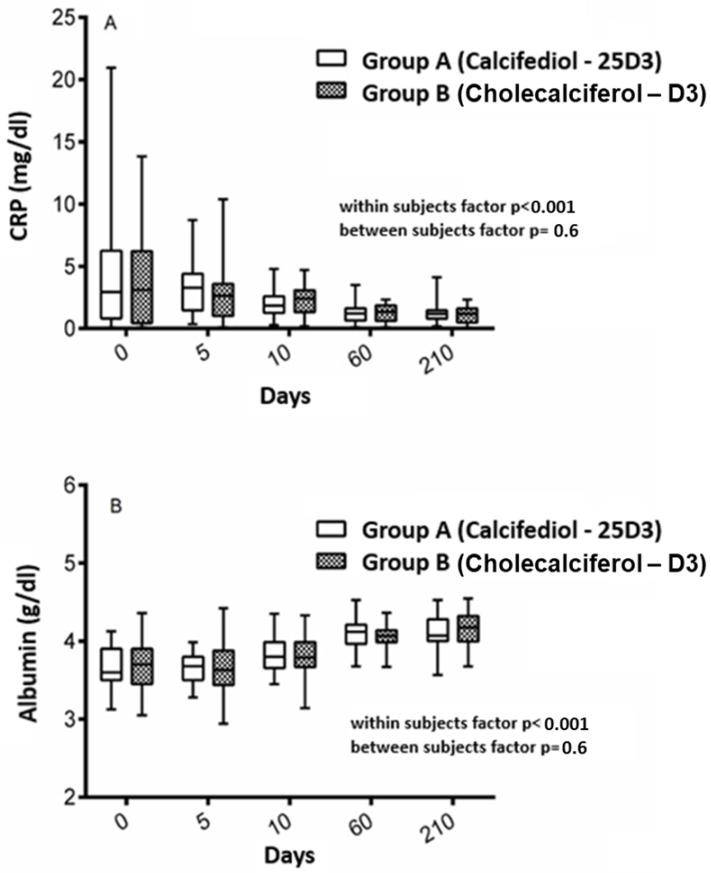
Serum levels of markers of inflammation and nutrition over time among participants grouped according to the type of intervention over time. Panel **A** shows the serum levels of CRP over time among participants belonging to group A, those receiving calcifediol, and group B, those receiving cholecalciferol. Panel **B** shows the serum levels of albumin over time among participants belonging to group A, those receiving calcifediol, and group B, those receiving cholecalciferol.

In both groups, CRP levels decreased over the study period from 5.1 ± 5.9 to 1.3 ± 0.9 mg/dl among participants belonging to group A, and from 3.9 ± 4.1 to 1.1 ± 0.7 mg/dl among those in group B (Figure 4, panel A). We estimated 0.5 mg/dl decline per month in group A and 0.4 mg/dl per month in group B, without difference between groups (*p* = 0.6), and by the group over time (time*intervention factor: *p* = 0.3). Similarly, albumin levels improved persistently over the study period without statistically significant differences between groups (Figure 4, panel B).

Ultimately, serum levels of CTx-I and BPA were almost superimposed within and between subjects over the entire study period (data not shown). Independent of several confounders, the baseline number of drugs and hand-grip strength were the main predictors of therapeutic success of intervention at the end of the study period. The higher the number of drugs, the lower the probability of therapeutic success was. Instead, the higher the hand grip strength, the higher the probability was to reach the therapeutic success of the intervention (Table 2). For instance, the probability that intervention being successful was higher in participants not taking any drugs compared to patients taking five drugs (83% and 63%, respectively), while it was about 19% among participants taking the maximum number of drugs (19 drugs). Consistently, the probability to achieve the therapeutic success of intervention ranged from 17% to 29% in women with low muscle strength (12 Kg and 17 Kg, respectively), reaching the threshold of 85% in women with the highest muscle strength (40 kg) (data not shown).

## 4. Discussion

This is a randomized controlled pragmatic trial investigating the effect of a single dosage of 25D3 and D3 supplementation in the oldest-old, hospitalized men and women, functionally preserved before admission. Compared to weekly supplementation of D3, a similar dose of 25D3 is associated with a faster increase over time of 25(OH)D, and a deeper decline of iPTH serum levels. However, the slopes of increasing 25(OH)D turned out to be similar between groups when differences of baseline iPTH levels were taken into account. Both 25D3 and D3 supplementations were associated with a similar increase of 1-25(OH)D, albumin, calcium and phosphorus serum levels, and a similar decline of CRP levels over time. Bone turnover markers did not show any statistically significant changes over time, independent of the type of intervention. Ultimately, we add to the current literature by showing that polypharmacy and low muscle strength may negatively affect the therapeutic success of the vitamin D supplementation, independent of the type of intervention, serum levels of bone and inflammatory markers at baseline.

Several authors highlighted that 25D3 acts faster, and is more potent than D3 in obtaining optimal serum levels of 25(OH)D, thus suggesting 25D3 may represent a satisfactory strategy to reach optimal 25(OH)D serum levels even in the oldest-old people. Administering D3 at a daily dose of 800–4000 IU allows to easily reach serum 25(OH)D target of 20 ng/mL, while it is more difficult to reach and maintain the target of 30 ng/mL, especially in obese and older adults with comorbidities [25]. The rates of older people with vitamin D inadequacy are liable to make the costs of supplementation increasingly higher, without hitting the object. Additional concerns are associated with D3 high doses (100,000 IU), causing an increased risk of hypercalciuria, hypercalcemia, bone turnover markers and fractures. From a pre-clinical perspective, the attainment of the optimal threshold of serum 25(OH)D might take advantage of different chemical properties of 25D3 compared to D3, and pharmacokinetics leading to a reduced risk of potential adverse effects [26]. However, a limited number of in vivo studies have evaluated the efficacy of 25D3 in increasing serum 25(OH)D concentrations compared with oral D3. Stamp et al. demonstrated that 25D3 seems to be about 10-fold more potent than D3 or D2 in increasing serum 25(OH)D concentrations [27]. However, the lack of homogeneity of the groups, the inclusion of subjects with metabolic bone diseases, the administration of both D3 and D2 in the intervention arm and differences in terms of treatment duration between groups hampered the validity of such findings. The ability of 25D3 to increase serum 25(OH)D more than D3 mainly stemmed from randomized double-blind controlled and prospective open-label trials using multiple doses of intervention, ranging from 5 to 20 mcg/day. To date, two randomized double-blind controlled and one prospective open-label trials tested the effects of a similar dose of 25D3 and D3 (20 mcg/day). Oldest-old people was constantly excluded from previous studies, which mainly enrolled post-menopausal women, occasionally with osteopenia, and tested daily administration of 25D3 and D3. Our study adds to the literature by showing the effects of 25D3 and D3 in the oldest-old people by using a weekly similar dosage, to overcome the issue of poor compliance related to polypharmacy.

Consistent with previous studies, our findings partially support the evidence that 25D3 acts faster than D3 to increase the serum levels of 25(OH)D over time in oldest-old people. Our study adds to the literature by suggesting an interaction factor between vitamin D supplementation and iPTH basal levels, so that the steeper slope of 25(OH)D serum levels associated with 25D3 might be driven by higher iPTH levels of participants on 25D3 intervention compared to those on D3. However, the sample size of our study does not allow expressing a conclusive judgment.

The trend of 25(OH)D serum levels was similar to that observed by Navarro-Valverde et al. [28] by using 20 mcg/day of 25D3 in post-menopausal women, 67 years old on average, and with mean baseline 25(OH)D levels of about 15 ng/mL. Likewise, findings from Jetter et al. showed that serum levels of 25(OH)D raised to the same extent of a 15 week trial by using the dosage of 140 mcg/weekly in 35 healthy post-menopausal women, aged 50-70 years, with baseline serum levels of 25(OH)D of about 13 ng/mL [29]. From a qualitative standpoint, the hospital stay was associated with a steeper increase of serum 25(OH)D compared to the trend shown in the outpatient setting, which may be explained by the full compliance of participants to the intervention during the former phase. A positive trend was also confirmed for serum levels of 1-25(OH)D, phosphorous, calcium and albumin independently from the type of vitamin D intervention. Likewise, findings from Cashman et al. showed that weekly supplementation of 25D3 was associated with an accelerated decrease in serum iPTH concentrations compared to D3, which in our study was particularly robust during days of hospital stay [30].

In addition, we showed a negative trend for CRP over the study period without differences between interventions. Several studies strongly demonstrated the link between low vitamin D levels, secondary higher levels of iPTH, higher inflammatory markers and impaired bone-muscle health [31]. Although in vivo and preclinical studies showed that vitamin D supplementation reduces iPTH and inflammatory markers among comorbid people, we acknowledge that the normalization of CRP observed in our study may be associated with the recovery of healthy status. Thus, we acknowledge that our study design, by choosing hospitalized oldest-old people, cannot permit to explore a direct effect of vitamin D supplementation on CRP as well as albumin levels. On the other side, the autocrine functions of 25D3 might still contribute to explain a stronger effect on iPTH and CRP compared to D3 [32].

Our group already showed that low vitamin D concentrations may be a surrogate of poor health and that comorbidity may exert a negative influence on the serum concentrations of vitamin D [33]. We add now that polypharmacy and low muscle strength are both negative predictors of the therapeutic success of the intervention, independently from type of intervention and several clinical characteristics. Polypharmacy, as proxy of comorbidities, may negatively interfere with the attainment of optimal serum levels of 25(OH)D by the means of several mechanisms, including reduced compliance and adherence to treatments, and interference with drug adsorption. Additionally, polypharmacy may reveal the need for a higher amount of vitamin D supplements or, alternatively, the existence of much more complex and integrated pathophysiological pathways to control vitamin D homeostasis. Low muscle strength, vitamin D and polypharmacy are all correlates of vulnerability and frailty among oldest-old people. Frailty is associated with multisystem alterations and dysregulations [34], which require multidimensional intervention to reach optimal serum levels of 25(OH)D, iPTH and CRP. Thus, a tailored approach should be considered to correct vitamin D inadequacy in oldest-old people, taking into account their multisystem dysregulations.

Ultimately, our findings confirm evidence about the pervasive vitamin D inadequacy among oldest-old people, by showing that 90.5% of participants had low 25(OH)D serum levels [1,35,36], despite being physically and cognitively preserved before hospitalization. About 37% of participants had vitamin D-insufficiency with secondary hyper-parathyroidism with calcium, phosphorus and creatinine levels in the normal range. Despite the significant increases in serum 25(OH)D concentrations, as already shown by Cashman [30] and Bischoff-Ferrari et al. [26], in our study, we add that 25D3 supplementation was not associated with hypercalcemia or alterations of bone turnover markers.

We acknowledge some limitations. This is a randomized pragmatic clinical trial based on hospitalized, oldest-old people moving from the in-patient to the outpatient setting, then respecting the rigorous design of randomized control trial in the former phase and highlighting the changes associated with real life setting in the second phase. In our study, we did not implement strategies to prove the participants’ level of compliance and adherence over time. Participants received telephone calls to make sure of compliance with schedules, with the type of intervention and adherence at the time of ambulatory follow-up. Participants’ physicians received a letter from investigators explaining the study design, intervention and procedures. We acknowledge the discrepancy of iPTH levels between groups. According to study design, randomization was performed blinding of serum biochemical markers at baseline, and we became aware of such a discrepancy at the time of data analysis. We argue that the accelerated decline of iPTH associated with an accelerated increase of 25(OH)D cannot be considered as a statistical artefact. There is plenty of biological plausibility for a direct effect of 25D3 on accessory pockets of vitamin D receptor activating genomic and non-genomic transcription [37]. In addition, calcium intake and/or calcium wasting may affect iPTH levels. As per protocol, during hospital stay participants received 500 mg calcium supplements twice a day, at discharge they received recommendations to assure adequate calcium intake from diet and, eventually, from supplements. Although in the study protocol we planned to collect 24-h urine, few participants contributed to urine collection due to several reasons, mainly urinary incontinence, mobility limitations or difficulties to reach bathroom during nighttime and nursing staff burden. Using data from 25 participants, we found low levels of 24 h-urine calcium and phosphorous in both groups.

We also recognize that the supplementation with 25D3 might influence the concentration of 24,25(OH)2D3, which is a catabolic form of 25(OH)D playing a biological role in the calcium homeostasis. Levels of 24,25(OH)2D3 may reduce the bioactivity of 25(OH)D and 1,25(OH)2D, particularly the extra-renal production of 1,25(OH)D, and down-regulate the secretion of iPTH [38]. Our study cannot add evidence about the role of 24,25(OH)2D in the interplay between 25(OH) D3, 1-25 (OH)2D3 and iPTH, given measures of 24,25(OH)2D3 are not available.

The strength of our study is the pragmatic design. Our findings may resemble the usual clinical practice by means of assessing the effects of already marketed medicines and making the findings applicable to multiple other settings. On the one hand, the blinding approach with respect to blood assays at the time of randomization assured casual distribution of eligible participants between groups. On the other hand, it caused the unbalanced distribution of participants with higher iPTH levels between groups. We believe that the unbalanced distribution of participants with higher iPTH levels did not weaken the main findings.

In conclusion, 25D3 supplementation seems to be effective as D3 among oldest-old people taking a similar dosage in multiple healthcare settings. The administration of 25D3 might be more beneficial in the oldest-old people as compared with D3 to rapidly increase serum levels and reach optimal target threshold, especially among those with comorbidity, taking multiple drugs and showing low muscle strength. At least in these specific conditions, 25D3 can be used as a favorable alternative to D3, with possibly lower cost per IU. However, clinical trials are still required to investigate the efficacy and safety of a similar amount of 25D3 and D3 for the treatment of osteoporosis and prevention of fragility fractures.

## Figures and Tables

**Table 1:** Baseline characteristics of participants according to with intervention group.

Characteristics	Group A	Group B	*p*-Value
Sex M-F (%)	12 (35)–22 (65)	13 (40)–20 (61)	0.9
Age (yrs)	83.5 (79.8–86.5)	82.0 (77.0–86.0)	0.2
BMI (Kg/m^2^)	26.8 (24.4–28.6)	27.5 (25.9–29.4)	0.1
ADL	5 (3–6)	5 (5–6)	0.8
IADL	4 (1–7)	4 (2–7)	0.6
CSHA-CFS	4 (3–5)	4 (3–5)	0.9
Hand grip, Kg			
Women	22.8 (18.6–24.6)	20.3 (17.1–29.8)	0.8
Men	28.8 (25.5–33.9)	28 (24.5–29.5)	0.3
MNA	24.5 (23.4-25.6)	24.0 (22.0–25.0)	0.2
MMSE	25 (23–27)	25 (22–28)	0.8
SBP (mmHg)	130 (120–146)	130 (119–140)	0.7
DBP (mmHg)	70 (60–80)	70 (60–80)	0.8
Heart rate (bpm)	75 (70–82)	70 (68–79)	0.3
Comorbidity (number)	7 (5–9)	7 (5–10)	0.6
Drugs (number)	6 (4–8)	5 (3–7)	0.2
CRP (mg/dl)	2.9 (0.8–6.3)	3.1 (0.5–6.2)	0.7
Creatinine (mg/dl)	0.9 (0.7–1.2)	0.8 (0.7–1.0)	0.1
iPTH (pg/mL)	104 (47–145)	50 (38–85)	0.002
H-iPTH (>85 pg/mL)	18 (52)	7 (21)	0.007
Calcium (mg/dl)	8.8 (8.5–9.1)	8.8 (8.6–9.5)	0.2
Phosphorus (mg/dl)	3.5 (2.9–3.6)	3.4 (2.9–4)	0.3
Magnesium (mg/dl)	2.0 (1.9–2.2)	2.1 (2.0–2.2)	0.3
CTx (ng/mL)	0.8 (0.5–1.2)	0.7 (0.4–1.0)	0.2
BAP (ng/mL)	9.1 (6.2–11.5)	9.2 (6.2–11.9)	0.9
25(OH)D (ng/mL)	10 (4–16)	10 (7–14)	0.7
25(OH)D deficiency n(%)	20 (59)	22 (66)	0.5
25(OH)D insufficiency n(%)	30 (88)	31 (93)	0.4
1.25(OH)_2_D (pg/mL)	27 (12–50)	22 (12–33)	0.4

Abbreviations: BMI (Body Mass Index), ADL (Activities Daily Living), IADL (Instrumental Activities of Daily Living), CSHA-CFS (Canadian Study of Health and Aging-Clinical Frailty Scale), MNA (Mini Nutritional Assessment), MMSE (Mini Mental State Examination), SBP (Systolic Blood Pressure), DBP (Diastolic Blood Pressure), CRP (C Reactive Protein), iPTH (Parathyroid Hormone intact), H-iPTH (Hyper-Parathyroidism), CTx (C-terminal telopeptide), BAP (bone alkaline phosphatase). Continuous and discrete data are summarized as median, 25^th^ and 75^th^ percentile; categorical data are expressed as number and percentage. Chi-square test or T-test as appropriate.

**Table 2:** Logistic regression models for the prediction of successful intervention.

Full Model	OR	95%C.I.	*p-*Value
**Sex**	0.9	0.2–4.8	0.9
**CSHA-CFS**	0.8	0.4–1.5	0.5
**Number of drugs**	0.7	0.5–0.9	0.02
**Hand grip**	1.2	1.0–1.4	0.02
**MNA**	1.1	0.9–1.5	0.3
**MMSE**	0.9	0.7–1.1	0.4
**ADL**	0.9	0.5–1.7	0.8
**IADL**	1.0	0.7–1.5	0.9
**Group B versus Group A**	0.4	0.1–1.5	0.2
**Restricted model** **(*p* < 0.25 in full model)**	**OR**	**95%C.I.**	***p-*value**
**Number of drugs**	0.7	0.6–0.9	0.01
**Hand grip**	1.2	1.0–1.3	0.01
**Group B versus Group A**	0.4	0.1–1.3	0.1

OR: Odds Ratio; 95%CI: Confidence Interval. Abbreviations: CSHA-CFS (Canadian Study of Health and Aging-Clinical Frailty Scale), ADL (Activities Daily Living), IADL (Instrumental Activities of Daily Living), MNA (Mini Nutritional Assessment), MMSE (Mini Mental State Examination).
